# Stimulation of Neurite Outgrowth Using Autologous NGF Bound at the Surface of a Fibrous Substrate

**DOI:** 10.3390/biom12010025

**Published:** 2021-12-24

**Authors:** Marta R. Casanova, Rui L. Reis, Albino Martins, Nuno M. Neves

**Affiliations:** 1 3B’s Research Group, I3Bs-Research Institute on Biomaterials, Biodegradables and Biomimetics of University of Minho, Headquarters of the European Institute of Excellence on Tissue Engineering and Regenerative Medicine, AvePark–Parque de Ciencia e Tecnologia, Zona Industrial da Gandra, 4805-017 Barco/Guimarães, Portugal; marta.casanova@i3bs.uminho.pt (M.R.C.); rgreis@i3bs.uminho.pt (R.L.R.); amartins@i3bs.uminho.pt (A.M.); 2 ICVS/3B’s–PT Government Associate Laboratory, 4805-017 Braga/Guimarães, Portugal

**Keywords:** biofunctionalization, electrospun fibrous mesh, nerve growth factor, neurite outgrowth, rat pheochromocytoma (PC12) cells

## Abstract

Peripheral nerve injury still remains a major clinical challenge, since the available solutions lead to dysfunctional nerve regeneration. Even though autologous nerve grafts are the gold standard, tissue engineered nerve guidance grafts are valid alternatives. Nerve growth factor (NGF) is the most potent neurotrophic factor. The development of a nerve guidance graft able to locally potentiate the interaction between injured neurons and autologous NGF would be a safer and more effective alternative to grafts that just release NGF. Herein, a biofunctional electrospun fibrous mesh (eFM) was developed through the selective retrieval of NGF from rat blood plasma. The neurite outgrowth induced by the eFM-NGF systems was assessed by culturing rat pheochromocytoma (PC12) cells for 7 days, without medium supplementation. The biological results showed that this NGF delivery system stimulates neuronal differentiation, enhancing the neurite growth more than the control condition.

## 1. Introduction

Nerve regeneration remains an enormous clinical challenge following peripheral nerve damage [1], which can drastically affect the quality of life of patients. With the rise of the number of accidents, peripheral nerve injury is becoming an increasingly common clinical problem. Although the peripheral nervous system has higher regeneration capacity than the central nervous system [2,3], the axonal reconnection and functional recovery by spontaneous repair is limited by the size and severity of the lesion [4,5].

Many treatment options could be used to repair nerve lesions, such as surgical reconnection and nerve allografts (autologous and alloplastic nerve grafts) [6,7,8,9,10,11,12], but the ideal treatment option for extensive nerve damage remains elusive (Table 1). The surgical reconnection by end-to-end suturing is commonly used to bridge small peripheral nerve injury gaps [7,9]. However, when the defect is large (>10 mm/>30 mm in rat/human) [13], a graft is required to support axonal regrowth, bridging the gap. Particularly, the use of autografts to bridge peripheral nerve gaps can lead to permanent donor site morbidity and inadequate functional repair [8,10,14]. Despite the improved treatment options applied in the clinic, the functional motor and sensory regeneration are frequently insufficient due to neuroma and scar formation, axonal loss, or target reinnervation failure [14,15]. Therefore, the recent advances in neural tissue engineering might be an alternative to conventional graft transplantation [16].

Tissue engineered nerve guidance grafts comprise a scaffold and a variety of cellular and/or molecular factors [11,13,18,19,20,21,22,23,24,25,26]. Usually, the scaffolds can guide the axonal regrowth and functional regeneration, restoring the nerve gap. Electrospun nanofibers were extensively studied as a potential scaffold in neural tissue engineering, due to the high specific surface area, its physical structure mimics the morphology of the native extracellular matrix, and the possible use as a carrier to deliver clinically relevant proteins like growth factors [25,27,28,29,30].

In order to improve nerve regeneration at the injury site, bioactive molecules are usually incorporated into the nerve grafts [19,24,25,26,29,31]. Among them, neurotrophic factors are an important group of bioactive molecules that stimulate and promote the proliferation of non-neuronal cells, as well as the sprouting/growth of injured sensory, motor, and postganglionic sympathetic axons from the proximal nerve stump [23,32]. Nerve growth factor (NGF) is one of the most prominent and commonly used neurotrophic factor for peripheral nerve regeneration [18,25,31,33,34,35,36,37]. It acts as a chemoattractant, which may regulate the proliferation and differentiation of cells, and the myelination of neurons [38]. Therefore, the development of nerve guidance grafts incorporating NGF is an emerging approach in the field of peripheral nerve regeneration [18,19,24,25,31,33,34,35,36,37]. Although encouraging, the NGF cost, its unstable clinical efficacy and uncontrolled release [20,31] leading to exposure to supraphysiological doses [39], has limited the clinical translation of these approaches. The use of autologous bioactive factors, avoiding immunogenicity, combined with the local delivery of bioactive autologous NGF presented at the surface of a biomaterial, avoids off-site adverse effects. Nevertheless, the amount of autologous bioactive factors is limited by the donor sampling, which requires always a collection process of the biological sample.

The immobilization of autologous bioactive molecules at the surface of medical device has aroused increasing interest. We herein validate for the first time the selective retrieval of NGF from rat blood plasma, bound at the surface of an electrospun fibrous mesh (eFM). The safety and efficacy of the eFM functionalized with autologous NGF (eFM-NGF) was validated in vitro, demonstrated by the neurogenic potency in enhancing neurite growth.

## 2. Materials and Methods

### 2.1. Rat Blood Plasma

Plasma derived from rat blood was collected from five 10-week-old adult male rats (Sprague Dawley, ENVIGO, Indianapolis, IN, United States), in accordance with the ethical and legal regulations (DGAV, n°021328 approved on 22 October 2020). The rats were housed in cages and had free access to water and food. After four weeks of acclimatization, at least, the animals were anesthetized by intraperitoneal injection of ketamine (75 mg/Kg) and medetomidine (0.5 mg/Kg), and the blood was collected by cardiac puncture. At the end of the cardiac puncture, animals were immediately euthanized by an overdose of pentobarbital sodium (200 mg/Kg) through an intracardiac injection. The blood plasma preparation was adapted from previously described methods [40]. Briefly, rat blood samples with anticoagulant (3.8% Citrate-dextrose solution; Sigma, Darmstadt, Germany) were incubated at 4 °C for 30 min. The blood samples were coagulated by the addition of calcium chloride (22 mM; Sigma, Darmstadt, Germany) and incubated at 37 °C for 40 min. Then, the clot was removed by centrifugation (890× *g* for 10 min) and the supernatant (plasma) kept at −80 °C until further use. A pool of rat blood plasma was done, and the neurotrophic factor concentration (i.e., NGFβ) assessed by the Rat beta-NGF DuoSet^®^ Enzyme-Linked Immunosorbent Assay (ELISA) (R&D Systems, Inc. a Bio-Techne Brand, Minneapolis, MN, USA), according to the manufacturer’s procedure. The pool of rat blood plasma was stored at –20 °C until further use.

### 2.2. Production of Electrospun Fibrous Meshes

The eFM were obtained by electrospinning a polymeric solution of 15% (*w*/*v*) Polycaprolactone (PCL-Mn 70,000–90,000 by GPC, Sigma-Aldrich, Darmstadt, Germany) in chloroform/dimethylformamide (7/3, Sigma-Aldrich, Darmstadt, Germany), by applying a voltage of 12 kV, a needle tip to ground collector distance of 20 cm, and a flow rate of 1mL/h [41]. After the processing of 1mL of the polymeric solution, the eFM was left to dry and cut into samples of 1cm^2^. The eFM is composed of fibers with diameters in the sub-micrometer range, from 0.4 to 1.4 μm, with an average pore size of 7.3 ± 3.2 μm and a thickness range from 40 to 60 μm [42,43].

The surfaces of the eFM were sterilized and activated by exposing both sides to UV-ozone irradiation (2 min; ProCleaner 220 system, Bioforce Nanoscience, Wetziar, Germany) and functionalized with amine groups (−NH_2_) by incubating in 1M 1,6-hexanediamine solution (Sigma, Darmstadt, Germany) for 1 h at 37 °C.

### 2.3. Nerve Growth Factor Functionalized Electrospun Fibrous Meshes

To determine the maximum immobilization capacity of the eFM, a wide range of the anti-NGF antibody concentrations (from 0 to 12 µg/mL) were used. The immobilization of the anti-NGF antibody (EP1320Y; Abcam) at the surface of the eFM was achieved using a covalent bond mediated by a coupling agent (1-ethyl-3-(3-(dimethylamino)-propyl)carbodiimide/hydroxysuccinimide mixture; Sigma, Darmstadt, Germany). After incubation for 2h at room temperature (RT), a bovine serum albumin (BSA; Sigma, Darmstadt, Germany) blocking step was performed (1h; RT), and the corresponding secondary antibody (1:200) (Alexa Fluor^®^ 488 donkey antirabbit IgG (H + L), Life Technologies, Bleuswijk, The Netherlands (~495/517 nm)) was incubated for 1 h (RT). To determine the degree of immobilization, the fluorescence of unbound secondary antibody solution was quantified (*n* = 3 samples, read in triplicate) in a microplate reader (Synergy HT, Bio-TEK, Winooski, VT, USA). Negative control samples consist of the substitution of the primary antibody immobilization step by 0.1 M phosphate-buffered saline (PBS; Sigma, Darmstadt, Germany). The samples were recovered to characterize the spatial distribution of the anti-NGF antibody by fluorescence microscopy (Axio Observer; Zeiss, Gottingen, Germany).

The eFM functionalized with anti-NGF antibody at the maximum concentration were incubated (1h; RT) with 200 μL of NGF from recombinant-origin (eFM-rNGF; 100 ng/mL rat recombinant NGF-β Sigma, Darmstadt, Germany) or from a pool of rat blood plasma (eFM-pNGF) as an autologous approach. The non-biofunctionalized eFM (eFM without anti-NGF antibody immobilization) was used as a negative control to evaluate the nonspecific binding of the NGF. All samples were rinsed with 0.1 M PBS (three washes; 5 min each). To assess the binding efficacy of the proposed delivery system, the unbound protein solutions (including washing solutions) were collected and stored at −20 °C, until further quantification by using the rat beta-NGF DuoSet^®^ ELISA kit (R&D Systems, Inc. a Bio-Techne Brand, Minneapolis, MN, USA). The NGF presented in the recombinant NGF solution or in the pool of rat blood plasma, the amount remaining after the immersion of the non-biofunctionalized and biofunctionalized eFMs, and also the amount of growth factor released in the washing solutions were all quantified to assess the binding capacity of the proposed delivery system.

### 2.4. Biological In Vitro Assays

The effectiveness of the developed eFM functionalized with NGFβ (Table 2), as a neurite outgrowth system, was assessed using rat PC12 cells derived from a pheochromocytoma of rat adrenal medulla. All the steps of eFM biofunctionalization were carried out with filtered solutions and under sterile conditions (inside a flow chamber). This cell line is a useful model system to study neuronal differentiation because the PC12 cells undergo differentiation when exposed to NGF. This cell line has been extensively used to evaluate biomaterials developed for nerve regeneration applications [29,30,44,45].

#### 2.4.1. PC12 Cells Culture

The rat PC12 cells were kindly provided by the Life and Health Sciences Research Institute (ICVS) of University of Minho. Cells were cultured in an expansion medium composed of DMEM medium (Sigma, Darmstadt, Germany) supplemented with 5% horse serum (HS; Sigma, Darmstadt, Germany), 10% fetal bovine serum (FBS; Life Technologies), and 1% antibiotic/antimycotic solution (ATB; Life Technologies) in a 75 cm^2^ cell culture flask coated with 50 μg/mL collagen type I from rat tail (Santa Cruz Biotechnology, Inc., Dallas, TX, USA). Cells were cultured at 37 °C in a humidified incubator with 5% CO_2_, and the medium was exchanged twice a week. For the assays, sub-confluent cells were harvested with TrypLE Express (1X) (Invitrogen, Bleiswijk, The Netherlands), seeded at a density of 20,000/eFM, and cultured in basal medium (BM: DMEM medium supplemented with 0.75% HS, 0.75% FBS, and 1% ATB). For the differentiation studies, PC12 cultured on eFM in BM supplemented with or without 100 ng/mL rat recombinant NGF-β (Sigma, Darmstadt, Germany) were carried out as positive or negative controls, respectively (Table 2). Cultures were incubated in a humid atmosphere at 37 °C and 5% CO_2_, and retrieved for further analysis at predefined culturing times, namely 1, 3, and 7 days. All tests were carried out in triplicate and repeated at least three times (*n* = 3), independently.

#### 2.4.2. Cellular Biochemistry Analysis

The cellular performance of PC12 on the different culture conditions was assessed for metabolic activity by the MTS assay (CellTiter 96 AQ_ueous_ One Solution; Promega, Madison, WI, USA), protein synthesis by Micro BCA assay (Micro BCA^TM^ Protein Assay Kit, Thermo Fisher Scientific, Bleiswijk, The Netherlands) and DNA quantification (Quant-iTPicoGreen dsDNA assay; Invitrogen, Bleiswijk, The Netherlands), according to the manufacturers’ instructions.

#### 2.4.3. Scanning Electron Microscopy (SEM)

The morphology of PC12 cells on the different culture conditions was analyzed by SEM (JSM-6010 LV, JEOL, Tokyo, Japan). Before SEM observation, specimens from all culture conditions were dehydrated and sputter coated with Au/Pd.

#### 2.4.4. Gene Expression Analysis

For the gene expression analysis, PC12 on different culture conditions, at each time point, were washed with PBS, immersed in Tri reagent^®^ (Life Science, VWR, Bleiswijk, The Netherlands), and kept at −80 °C until further use. Isolation of RNA was performed according to the Tri reagent^®^ instructions. The cDNA was amplified from 100 ng of total RNA through the qScript cDNA synthesis kit (Quanta Biosciences, VWR, Bleiswijk, The Netherlands). The qPCR reactions were carried out in a Mastercycler^®^ ep Gradient S realplex^®^ thermocycler (Eppendorf; Hamburg, Germany) for neurogenic genes (Table 3), according to the manufacturer’s instructions of the PerfeCtaTM SYBR^®^ Green system (Quanta Biosciences, VWR, Bleiswijk, The Netherlands). The housekeeping gene *Glyceraldehydes-3-phosphate-dehydrogenase* (GAPDH) was used to normalize the transcripts expression, and their quantification was achieved through the Livak method (2 ^−ΔΔCT^ method), considering the negative control as calibrator.

#### 2.4.5. Immunocytochemistry

Following the previously described cell culture conditions, samples were collected at 7 days fixed in a 10% formalin solution and kept at 4 °C until further used for staining procedures. Samples were incubated with beta III Tubulin (anti-beta III Tubulin polyclonal antibody, 1:100 dilution; Abcam, Cambridge, UK), GAP-43 (rabbit GAP43 polyclonal antibody, 1:200 dilution; Thermo Fisher Scientific, Bleiswijk, The Netherlands), NF200 (mouse anti-neurofilament 200 monoclonal antibody, 1:40 dilution; Sigma, Darmstadt, Germany) and Synapsin 1 (rabbit anti-synapsin I polyclonal antibody, 1:200 dilution; Abcam; Cambridge, UK) overnight at 4 °C, in a humidified atmosphere after the quenching of endogenous peroxidase activity (0.3% hydrogen peroxide solution; 30 min). R.T.U. VECTASTAIN^®^ Universal ABC Elite^®^ Kit (Vector Laboratories, Burlingame, CA, USA) was used for secondary antibody detection and incubation revealed by using the Peroxidase Substrate Kit (DAB) (Vector Laboratories, Burlingame, CA, USA), according to the manufacturer instructions. The samples were counterstained with hematoxylin for nuclei visualization, mounted in aqueous mounting medium (Sigma, Darmstadt, Germany), and observed in an optical microscope (Leica DM750 microscope).

#### 2.4.6. Neurite Outgrowth

Since PC12 cells differentiate towards the neuronal phenotype in response to NGF stimulation, the bioactivity of NGF from recombinant- (rNGF) or rat blood plasma (pNGF) origin immobilized at the surface of eFM was quantified by counting the neurite outgrowth. For different cell culture conditions (Table 2), at the different times points, the percentage of neurite-bearing cells was determined by counting beta III tubulin expressing PC12 cells (more than 200 cells in randomly selected fields (6–8), scanned from left to right, recorded at 40× lens objective (Leica DM750 microscope) with a computerized image analyzer (NIH ImageJ software, 2.1.0/1.53c, Bethesda, MD, USA). A PC12 cell was considered as neurite-bearing if containing at least one neuronal process. In cell clusters, the neurite-bearing cells are those with neurites length >2 μm from the cell body/nuclei staining [44,46] (Supplementary Figure S1).

### 2.5. Statistical Analysis

All statistical analysis was carried out by the SPSS statistical software (Version 26 for Mac). The Shapiro–Wilk test was performed to determine the data normality and the Levene test was carried out to check the homogeneity of variances. The normality and variance homogeneity were rejected, thus nonparametric tests were performed (Kruskal–Wallis test followed by Tukey’s HSD test). p values lower than 0.01 were statistically significant, being the confidence interval of 99%.

## 3. Results

### 3.1. Physicochemical Properties of the eFM Functionalized with NGF

**Figure 1:**
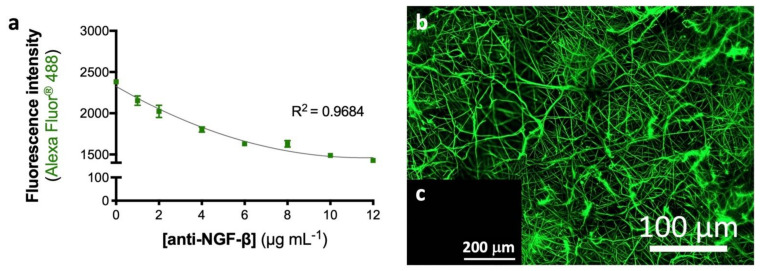
Activated and biofunctionalized eFM. Maximum immobilization capacity of anti-NGF-β antibody at the eFM surface (**a**). The one-way ANOVA with the Tukey’s HSD post hoc test were applied (*p* < 0.01): *a* represents significant differences compared to concentration 0 μg/mL; *b* represents significant differences compared to concentration 1 μg/mL; *c* represents significant differences compared to concentration 2 μg/mL; *d* represents significant differences compared to concentration 4 μg/mL; *e* represents significant differences compared to concentration 6 μg/mL and *f* represents significant differences compared to concentration 8 μg/mL. Spatial distribution of 10 μg/mL anti-NGF immobilized at the eFM surface (**b**). The negative control sample was not incubated with the primary antibody (**c**).

An optimized activated and functionalized eFM with anti-NGF antibody was developed, providing binding sites to NGF via a non-neutralizing antibody, envisioning an autologous approach. The maximum antibody immobilization capacity was determined using an indirect quantification method (Figure 1a). The antibody against NGF was immobilized at the surface of activated and functionalized eFM in a wide range of concentrations (0–12 µg/mL). The amount of unbound secondary antibody, after its incubation with the eFM immobilizing the primary antibody, was determined by fluorescence measurement. As observed in Figure 1a, higher amounts of immobilized anti-NGF antibody correspond to a lower fluorescence signal of the unbound secondary antibody. When the fluorescence signal reaches a plateau, the eFM presents the maximum concentration of immobilized primary antibody, reaching the saturation point of the system. The maximum concentration of immobilized anti-NGF antibody was achieved at 10 µg/mL, corresponding to a lower fluorescence signal of the free secondary antibody. The fluorescence micrograph (Figure 1b) shows a uniform distribution of anti-NGF antibody immobilized at the surface of the eFM. No fluorescence was detected in the control experiment in which all steps were performed except the incubation with the primary antibody (Figure 1c), evidencing a strong specificity of the primary antibody binding.

The binding capacity of the immobilized anti-NGF antibody was assessed by using NGF from recombinant-origin (rNGF) or from a pool of rat blood plasma (pNGF). The non-biofunctionalized eFMs (eFMs without anti-NGF antibody immobilization) were used as a negative control to evaluate the nonspecific binding of NGF. Table 4 shows that NGF from different origins was bound to the surface of eFM. Despite the different NGF concentrations, the binding efficiency stayed in the same range for the two different origins (approximately 95%), being 98 700 ± 200 pg/mL of rNGF or 567 ± 23 pg/mL of pNGF bound to the eFM biofunctionalized with anti-NGF.

### 3.2. Performance of PC12 Cells Cultured on eFM-NGF

PC12 cells were cultured over eFM functionalized with NGF from different sources (i.e., rNGF and pNGF), under basal conditions, to determine the effectiveness of these biofunctionalized systems.

**Figure 2:**
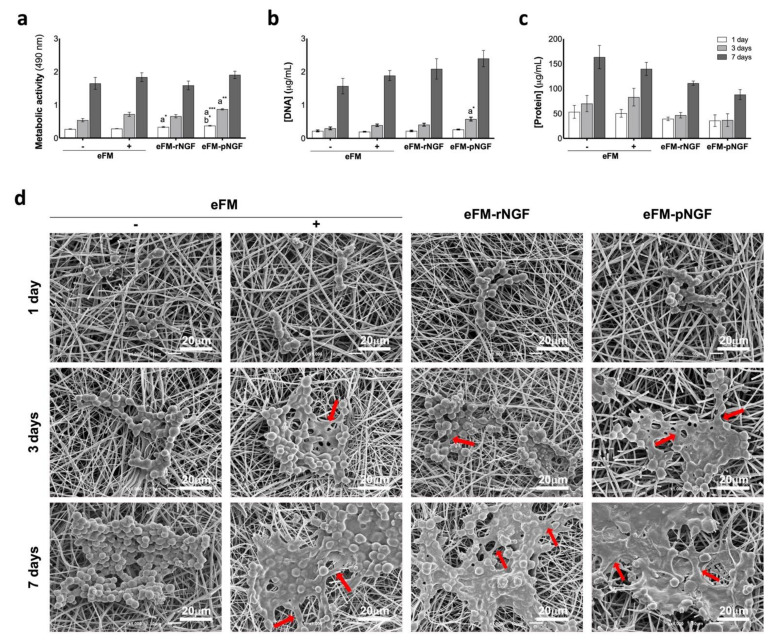
Biochemical performance of PC12 cells. (Top): Biochemical performance (i.e., metabolic activity (**a**), proliferation (**b**), and protein synthesis (**c**) of the PC12 cells cultured on eFM functionalized with NGFβ from different sources, under basal medium and at defined time points (1,3 and 7 days). eFMs cultured with PC12 cells under basal (eFM−) or neuronal differentiation media (eFM+) were used as controls. The one-way ANOVA with the Tukey’s HSD post hoc test were applied (*p* < 0.01): *a* represents significant differences compared to eFM− and *b* represents significant differences compared to eFM+; * *p* < 0.01; ** *p* < 0.001; *** *p* < 0.0001; (Bottom): Morphological analysis of the PC12 cells on different culture condition, along culturing time, by scanning electron microscopy (SEM) (**d**).

In terms of cell viability (Figure 2a), the PC12 cells cultured on the eFM comprising autologous NGF (eFM-pNGF) displayed significantly higher viability than the control conditions (eFM− and eFM+) at days 1 and 3 (*p* < 0.001). However, on the 7th day of PC12 culture, all cultured conditions have similarly high cell viability. The eFM-NGF systems were favorable for cell proliferation and protein synthesis since the levels of DNA content (Figure 2b) and the protein synthesis (Figure 2c) are comparable to those observed on the culture control conditions (eFM− and eFM+). As displayed in Figure 2d), PC12 cells attach, spread, and proliferate on all the eFM conditions. The morphology changes of PC12 cells culture on the different culture conditions along the culturing time reflect their differentiation stage. In the negative control condition (eFM−), the PC12 cells showed the morphology of undifferentiated cells, being lower and spherical on all culturing times. Although, in the presence of NGF (eFM+; eFM-rNGF; eFM-pNGF), the PC12 cells start to differentiate showing an elongated morphology. Indeed, a few cells grown on eFM without NGF treatment (eFM−) showing elongated shape, while on the eFM-pNGF system the elongated cell morphologies are consistently observed.

**Figure 3:**
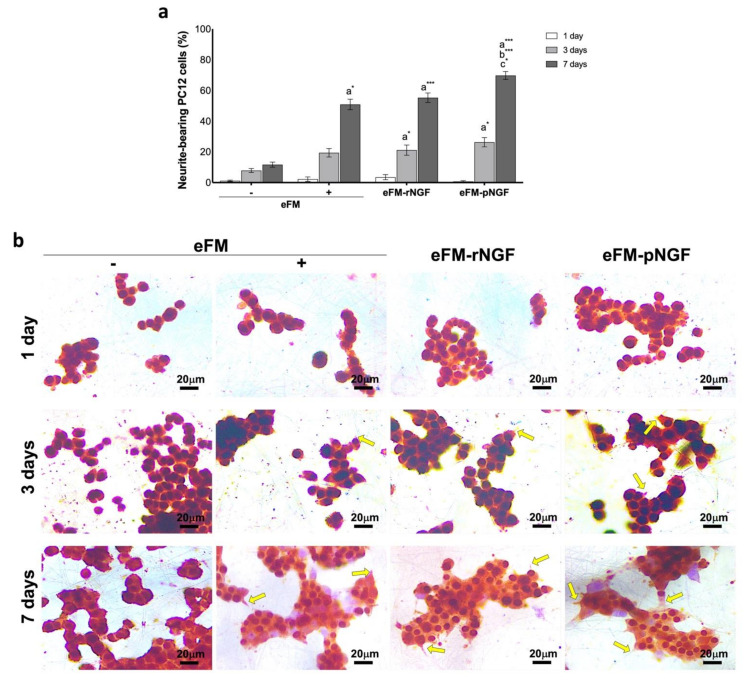
Neurite-bearing PC12 cells. (Top) Percentage of neurite-bearing PC12 cells on the different culture conditions along culturing time (**a**). The one-way ANOVA with the Tukey’s HSD post hoc test were applied (*p* < 0.01): *a* represents significant differences compared to eFM−; *b* represents significant differences compared to eFM+, and *c* represents significant differences compared to eFM-rNGF; * *p* < 0.01; *** *p* < 0.0001; (Bottom) Immunocytochemistry with beta-III Tubulin (brown) of PC12 cells on different culture condition along culturing time (**b**).

PC12 cell differentiation was studied by analyzing the number of neurite-bearing cells (differentiated PC12 cells), along the culturing time. After the 7th day of culture, the neurite outgrowth in the eFM-rNGF condition was comparable to the positive control condition (eFM+) (Figure 3a). Nevertheless, the neurite outgrowth in eFM-pNGF condition was significantly higher than that observed in either the positive or negative controls (eFM+; eFM−). The result suggested that the NGF bound at the eFM surface kept its bioactive, i.e., its ability to promote neurite outgrowth of PC12 cells (Figure 3b).

**Figure 4:**
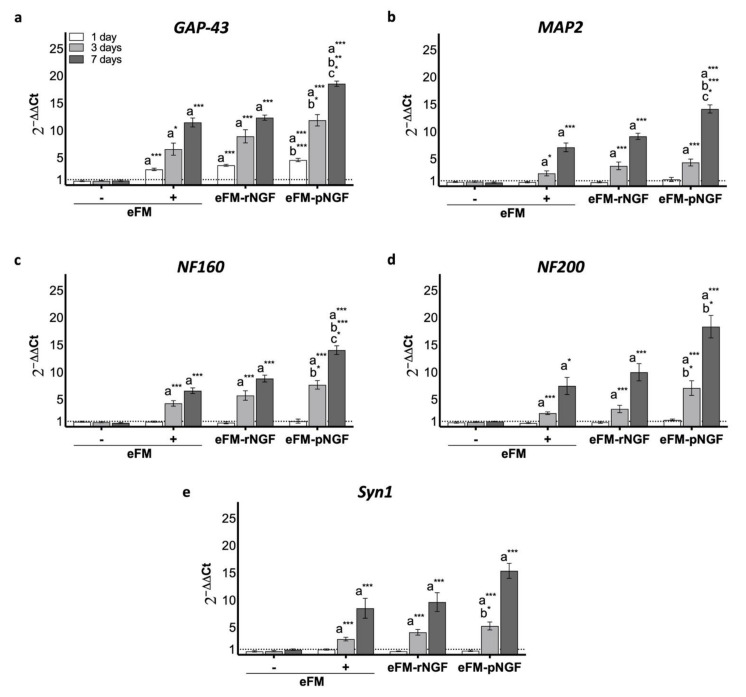
Relative expression of *GAP-43* (**a**), *MAP2* (**b**), *NF160* (**c**), *NF200* (**d**) and *Syn1* (**e**) genes by PC12 cells cultured on the different culture condition, along culturing time. The expression was normalized against the *GAPDH* gene, being the quantification performed with the negative control (eFM−) as calibrator, according to the Livak method. The one-way ANOVA with the Tukey’s HSD post hoc test were applied (*p* < 0.01): *a* represents significant differences compared to eFM−; *b* represents significant differences compared to eFM+, and *c* represents significant differences compared to eFM-rNGF; * *p* < 0.01; ** *p* < 0.001; *** *p* < 0.0001.

In order to further quantify the impact of eFM-NGF systems on PC12 cells differentiation, qPCR was used to analyze the expression levels of the *Growth-associated protein 43* regulation gene (*GAP-43*), the *Microtubule-associated protein 2* regulation gene (*MAP2*), the *Medium-molecular-weight neurofilament protein* regulation gene (*NF160*), the *High-molecular-weight neurofilament protein* regulation gene (*NF200*) and *Synapsin 1* regulation gene (*Syn1*). Except for *GAP-43*, all other genes are not expressed on the 1^st^ day of PC12 cells culture, in all testing conditions (Figure 4). On the 3rd day, PC12 cells cultured on the eFM-pNGF condition present significantly higher *GAP-43*, *NF160*, *NF200,* and *Syn1* expression when compared to positive control condition (eFM+) (*p* < 0.01). Likewise, the eFM-pNGF condition shown a significantly higher *GAP-43*, *NF160*, *NF200*, and *MAP2* expression than the eFM+ condition at 7 days of PC12 cell culture (*p* < 0.0001). Moreover, the eFM-pNGF condition showed significantly higher expression of *GAP-43*, *MAP2*, and *NF160* genes when compared to the eFM-rNGF condition (*p* < 0.01). These results indicate that the eFM-pNGF system may provide a stronger stimulation for the PC12 cell differentiation than the other conditions.

**Figure 5:**
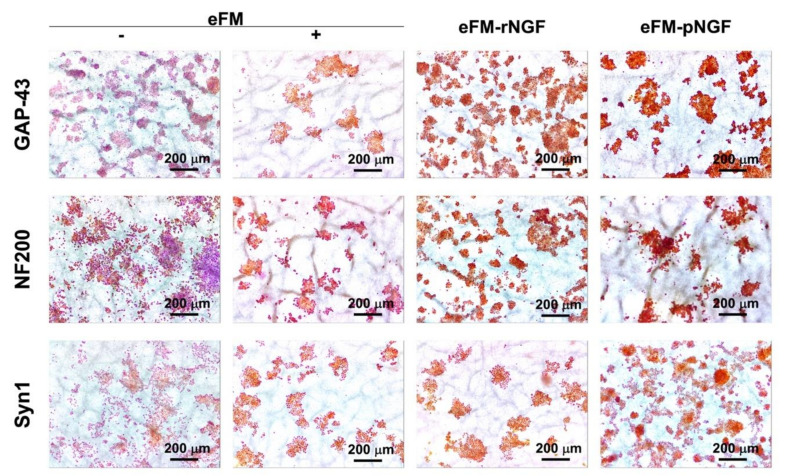
Immunoexpression of GAP-43, NF-200, and Syn1 proteins by the PC12 cells cultured on the different conditions on the 7th day.

After 7 days of culture, the PC12 cells were immunostained for GAP-43, NF200, and Syn1. Figure 5 demonstrated that NGF-stimulated differentiation of PC12 cells toward the neural cell phenotype in eFM+ condition was similar to, although a slightly lower than, the eFM-pNGF or eFM-rNGF conditions (Supplementary Figure S2). Furthermore, neuronal differentiation in both eFM-NGF systems was significantly higher than in the negative control condition (eFM−). Collectively, these phenotypic results confirm the previously described genotypic results.

## 4. Discussion

Nerve regeneration comprises neuronal growth and the development of the extracellular matrix [5,45]. Nerve guidance grafts aspire to promote a regenerative microenvironment that enable axonal regrowth and, simultaneously, confines the scar formation [16,23]. Despite the extensive knowledge on the pathophysiology and the regeneration mechanisms of peripheral nerve injury, reliable treatment options that ensure full functional recovery are still not available. Thus, in this work, we developed a neurogenic-inductive system through the immobilization of autologous NGF at the surface of a substrate, capable to promote axonal outgrowth in vitro as a reliable treatment option.

The present study aims to study the neurogenic potential of eFM functionalized with autologous NGF via the antibody-antigen binding. Herein a non-neutralizing antibody against NGF was successfully immobilized at the surface of activated and functionalized eFM, achieving the maximum immobilization capacity at 10 μg/mL. Early studies reported that the immobilization capacity of electrospun nanofibrous substrates differ among antibodies, namely 12 μg/mL for anti-TGFβ1, 8 μg/mL for anti-bFGF, 6 μg/mL for anti-TNF-α and 4 μg/mL for anti-VEGF, anti-TGFβ3 and anti-IGF-I [41,47,48,49,50,51].

Platelet-rich plasma has been used to stimulate tissue regeneration, especially in peripheral nerve, with growing evidence at the research and clinical levels [52,53,54,55]. Plasma-derived NGF levels in healthy human patients (from blood samples obtained by venipuncture) vary between 100–752 pg/mL [56,57,58], while in healthy rats varies between 400–1000 pg/mL [59,60,61]. Considering these values, the amount of NFG in a pool of rat blood plasma presented similar concentration (597 ± 40 pg/mL) to the ones reported in the literature. Further on, the bioactivity of the anti-NGF immobilized at the eFM surface, for the selective retrieval of the corresponding growth factor, was evaluated by using a biological fluid (i.e., rat blood plasma) and a recombinant NGF (rNGF). The developed biofunctional eFM displayed a high binding efficiency (approximately 95%) for both sources.

PC12 cells are extensively used as a model to study neuronal cell differentiation through its neurite outgrowth capacity [29,30,34,44,45,48]. They can be triggered by NGF to differentiate into neuron-like cells, forming myelin structures. Undifferentiated PC12 are a type of semi-suspended and aggregate cells with weak sensing toward the culturing substrates. The proliferation rate and viability of PC12 cells were favorable on the eFM-NGF systems. Moreover, the neurite outgrowth was significantly enhanced on the eFM-NGF systems, especially on the eFM-pNGF system. These results were consistent with recent studies where electrospun nanofibrous scaffolds releasing NGF enhanced neural cell commitment [33,34,35,36,37]. However, the stimulation of neurite outgrowth and neural differentiation on different neurotrophic factor-containing scaffolds varied between works. In general, the neurite outgrowth is neurotrophic factor dose-dependent with optimal concentrations in the range of 1–10 ng/mL. Herein, the autologous NGF bound at the surface of eFM (eFM-NGF system) increased neuronal viability and induced differentiation with enhanced neurite outgrowth. These results were further confirmed by analyzing the expression of neural markers (*GAP-43*, *MAP2*, *NF160*, *NF200*, and *Syn1*) by qPCR and immunochemistry, which confirmed the prior observation that the eFM-NGF systems induce differentiation of PC12 cells into neuron-like cells. Therefore, our immobilization strategy guarantees the bioactivity of bound NGF, extending the half-life of the protein when compared to its free form, as confirmed by the direct culture of PC12 cells over the biofunctionalized eFM systems (eFM-rNGF and eFM-pNGF).

The herein developed neurogenic-inductive biofunctional system, consisting on the immobilization of anti-NGF antibody at the surface of eFM, allows the selective retrieval of autologous NGF derived from rat blood plasma. Thus, the novelty of this approach relies on the implementation of very effective and personalized therapies tailored for specific clinical conditions, by using biological fluids from an autologous source. However, in light of the results achieved, a complementary in vivo study in a rat nerve defect model is needed to validate the efficacy of this neurogenic-inductive system.

## 5. Conclusions

The eFM-pNGF system promotes axonal outgrowth, exhibiting a higher potential to differentiate PC12 cells towards the neurogenic lineage, as compared to the eFM-rNGF system. Interestingly, the results showed that our envisioned autologous approach (eFM-rNGF system) is more effective in promoting neurite extension and outgrowth, avoiding the use of medium supplementation. Therefore, the herein proposed neurogenic-inductive eFM system might have an application in peripheral nerve tissue engineering, which is an unmet clinical need.

This study demonstrate the in vitro safety and efficacy of the eFM-NGF systems comprising NGF from different sources (recombinant or rat blood plasma) to enhance the stimulation of neurite outgrowth. Cell culture assays with PC12 cells demonstrated that the developed neurogenic-inductive eFM system can successfully promote neurogenic differentiation, displaying typical phenotypic and genotypic markers. Moreover, the eFM functionalized with autologous NGF (eFM-pNGF system) can enhance the stimulation of neurite outgrowth, being more effective than the positive control condition, without the need for a continuous supply of exogenous recombinant growth factors. We hypothesize that our system maximizes the exposure of the PC12 cells to the immobilized NGF, by the close contact established between them and the NGF bound at the surface of the fibrous substrate. Therefore, the proposed eFM-pNGF system represents a promising medical device to locally stimulate neurite outgrowth.

## 6. Patents

This work was developed under the scope of the provisional patent application nr. 116,970 “*Polymeric substrates for nerve regeneration, methods and uses thereof*”, priority date 3 December 2020.

## Figures and Tables

**Table 1:** Strategies applied in nerve regeneration, underlining the pros and cons ^a^.

Strategies		Pros	Cons
Nerve Grafts	Autograft	− Absence of immunological rejection; − Provides a suitable environment for nerve regeneration;	− Donor site morbidity and potential loss of function; − Limited number of grafts;
	Allograft	− Unlimited source tissue; − No donor site trauma;	− Uncertain histocompatibility; − Ethical and legal concerns;
	Xenograft	− Large number of donors; − Similar physical, chemical, and mechanical properties of the injured nerve;	− Immune rejection; − Treatment of recipients with immunosuppressive drugs;
Tissue Engineered Nerve Guidance Grafts		− Produced from polymers or biomacromolecules; − Unlimited source materials; − No donor site trauma;	− Antigenicity; − Difficult to suture to the injured nerve; − Poor environment for nerve regeneration;

^a^ Adapted from Gao Y, et al., 2015 [17].

**Table 2:** Experimental conditions used in the cell biology assays.

Condition		Description
eFM	−	eFM in BM ^1^ without rNGF
	+	eFM in BM supplemented with 100 ng/mL of rNGF
eFM-rNGF		eFM functionalized with rNGF in BM
eFM-pNGF		eFM functionalized with plasma-derived NGF in BM

^1^ BM: DMEM supplemented with 0.75% horse serum (HS), 0.75% fetal bovine serum (FBS), 1% antibiotic/antimycotic (ATB).

**Table 3:** Primer sequences used for RT-PCR procedures ^1^.

Gene ^1^	Forward (5′-3′)	Reverse (5′-3′)
*GAPDH*	CAACTCCCTCAAGATTGTCAGCAA	GGCATGGACTGTGGTCATGA
*GAP-43*	TTTCCTCTCCTGTCCTGCTC	TGGACTTGGGATCTTTCCTG
*MAP2*	GGCACTCCTCCAAGCTACTCT	CTTGACGTTCTTCAGGTCTGG
*NF-160*	AGCATTGAGCTCGAGTCGGTG	CTGCTGGATGGTGTCCTGGTAG
*NF-200*	AAAGTGAACACGGATGCTATGC	GTGCTTTTCAGTGCCTCCAAC
*Syn1*	GTGTCAGGGAACTGGAAGACC	AGGAGCCCACCACCTCAATA

^1^ GAPDH: *Glyceraldehyde 3-phosphate dehydrogenase*; GAP-43: *Growth-associated protein 43*; MAP-2: *Microtubule-associated protein 2*; NF-160: *Neurofilament 160*; NF-200: *Neurofilament 200*; Syn1: *Synapsin-1*.

**Table 4:** Quantification of NGF recombinant or derived from a pool of rat blood plasma, and the binding capacity of the eFM-NGF system.

	Recombinant (ng/mL)	Plasma (ρg/mL)
[NGF]	99.9 ± 0.01	597 ± 40
bound	98.7 ± 0.2	567 ± 23

## Data Availability

The data that support the findings of this study are available from the corresponding author upon reasonable request.

## Supplementary Materials

The following are available online at https://www.mdpi.com/article/10.3390/biom12010025/s1, Figure S1: Neurite-bearing PC12 cells, Figure S2: Protein expression, namely GAP-43, NF200 and Syn1 represented by DAB staining intensity normalized by nucleus from color deconvolution analysis.
