# Supplementary material for: Stimulation of Neurite Outgrowth Using Autologous NGF Bound at the Surface of a Fibrous Substrate
Source: Biomolecules. 2021 Dec 24;12(1):25. doi: 10.3390/biom12010025 (PMC8773656; doi:10.3390/biom12010025)
Supplement: Supplementary file 1 [file biomolecules-12-00025-s001.zip › biomolecules-1492784-supplementary.pdf]

Supplementary Materials

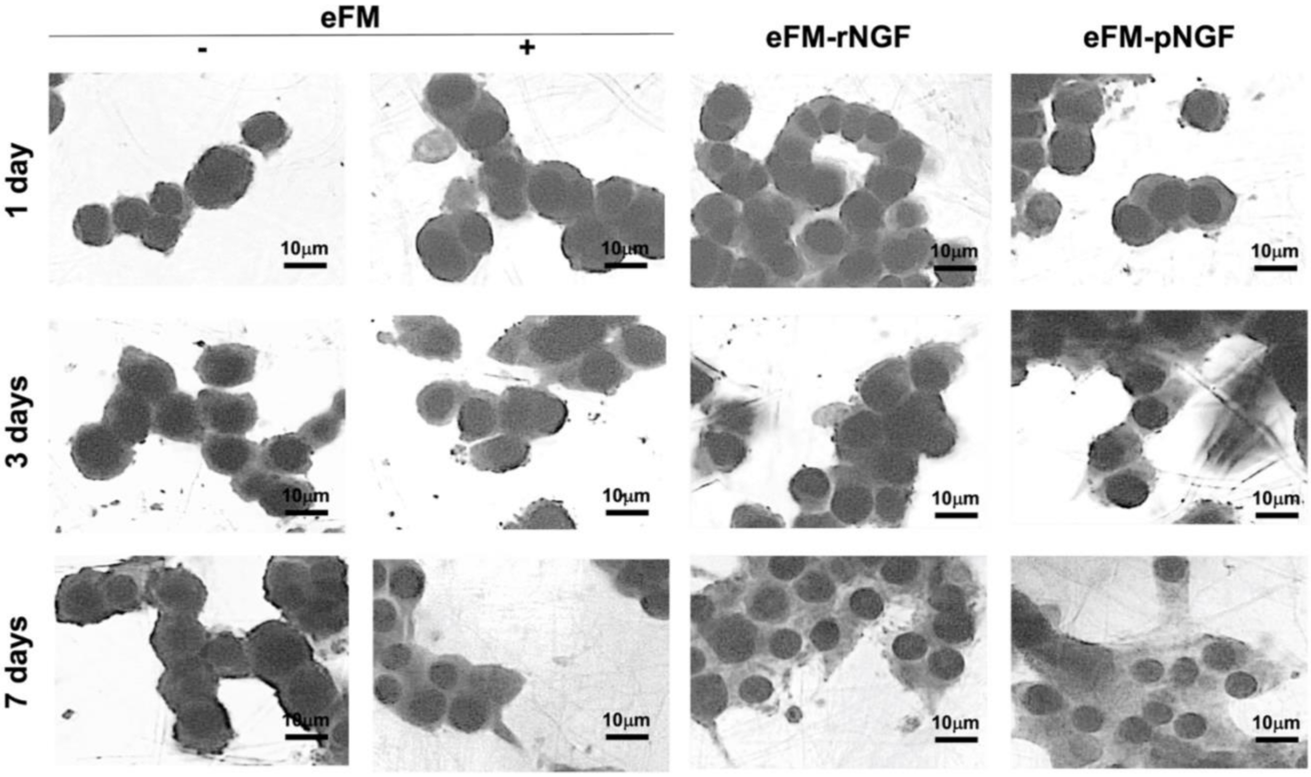

Figure S1. Neurite-bearing PC12 cells.

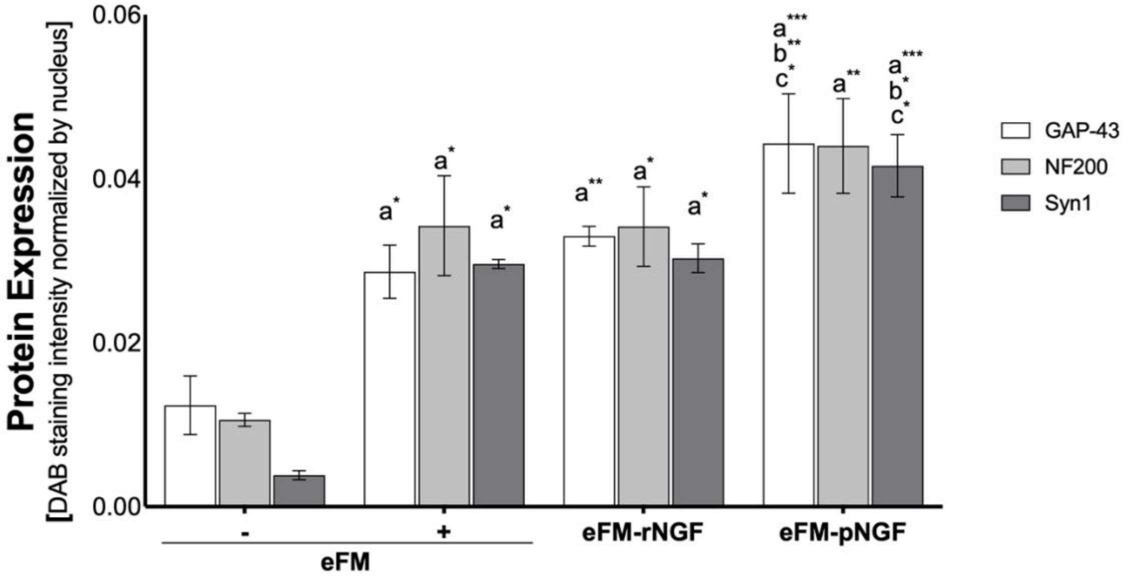

Figure S2. Protein expression, namely GAP-43, NF200 and Syn1 represented by DAB staining intensity normalized by nucleus from color deconvolution analysis. The one-way ANOVA with the Fisher's post hoc test were applied ($p<0.01$): a represents significant differences compared to eFM-; b represents significant differences compared to eFM+, and c represents significant differences compared to eFM-rNGF; *$p < 0.01$; **$p< 0.001$; ***$p< 0.0001$;
